# Preparing for later life in the Oosterpark neighborhood: a mixed-methods study among middle-aged and older adults

**DOI:** 10.1186/s12889-026-27350-z

**Published:** 2026-06-17

**Authors:** Nanda Kleinenberg-Talsma, Fons van der Lucht, Herbert Rolden, Harriët Jager-Wittenaar, Evelyn Finnema

**Affiliations:** 1 Research Group Healthy AgeingAllied Health Care and Nursing, Hanze University of Applied Sciences Groningen, Petrus Driessenstraat, Groningen, 3, 9714 CA The Netherlands; 2 University of Groningen, University Medical Center Groningen, Research Institute Health in Context, Hanzeplein 1, Groningen, 9713 GZ The Netherlands; 3 FAITH research, Groningen/Leeuwarden, The Netherlands; 4 National Institute of Public Health and the Environment, Centre for Health and Society, Bilthoven, The Netherlands; 5 Aletta Jacobs School of Public Health, Groningen, The Netherlands; 6 Department of Gastroenterology and Hepatology, Radboud University Medical Center, Dietetics, Nijmegen, The Netherlands; 7 Faculty of Physical Education and Physiotherapy, Department Physiotherapy and Human Anatomy, Research Unit Experimental Anatomy, Vrije Universiteit Brussel, Brussels, Belgium; 8 Department of Health Science, Section of Nursing Research, University of Groningen, University Medical Center Groningen, Groningen, The Netherlands; 9 Research Group Nursing Diagnostics, Hanze University of Applied Sciences Groningen, Groningen, The Netherlands; 10 Research Group Living, Wellbeing and Care for Older People, NHL Stenden University of Applied Sciences, Leeuwarden, The Netherlands

**Keywords:** Age-related preparation, Aging, Frailty, Mixed-methods research, Pillar Integration Process, Prevention, Public health

## Abstract

**Background:**

The global population is aging, and with this development, frailty prevalence is increasing as well. Age-related preparation activities could benefit the prevention or delay of frailty. Local stakeholders such as municipalities, welfare organizations, and community health services should understand how to support (future) older adults in old-age preparations in their local context. This study aims to describe and understand middle-aged and older adults’ focus points, needs, and wishes in preparation for older age.

**Methods:**

A mixed-methods study was conducted in the Oosterpark neighborhood in Groningen, a city in northern Netherlands, in 2023. Questionnaires regarding age-related preparations were collected both digitally and on paper. The questionnaire addressed nine themes: financial situation, emergencies, mental and physical fitness, housing, looks and appearance, personal relationships, health, leisure activities, professional activities. Additionally, focus group discussions were organized in the community center. Data were analyzed separately in SPSS and ATLAS.ti, and integrated analysis was conducted following the Pillar Integration Process.

**Results:**

In total, 134 questionnaires were completed (mean age 59 ± 12 years), six focus groups and two individual interviews were conducted. While respondents scored high on preparation for themes as physical and mental fitness or personal relationships, scores for themes as housing and leisure activities were lower. Participants identified a tenth theme, *neighborhood*, and several subthemes were added to existing themes of health and personal relationships. Although many were concerned with age-related preparation, few engaged in active preparation. People also indicated not knowing how to prepare or where to request help.

**Conclusion:**

The study identified that middle-aged and older adults think about age-related preparation often, but hardly prepare actively for themes such as housing or financial situation. Local stakeholders, e.g., municipalities, social welfare organizations and community health organizations, should stimulate, facilitate and support citizens to prepare for later life by offering practical support, e.g. easily accessible information points. Taking into account the physical and social living environment of older adults is crucial. Better age-related preparedness could help to prevent or delay frailty, prolong independence and increase quality of life.

**Supplementary Information:**

The online version contains supplementary material available at https://doi.org/10.1186/s12889-026-27350-z.

## Background

Due to different demographic trends, such as increased life expectancy and lower birth rates, the global population is aging [1]. With an increase in the number of older adults, the health system, public health, and society are under more pressure. As people age, they increasingly need health and social care services and are more at risk of becoming frail [2]. Frailty is a common condition in older adults, defined as a decline in functioning in one or several health domains, increasing one’s vulnerability to stressors [3].

Regarding frailty prevention and aging well, the role and responsibility of older adults is growing. In many Western welfare states, the level and breadth of services and care provision are diminishing, and for support and care, there is an increasing demand on individuals and their networks [4, 5]. The Netherlands is an example of a welfare state in which social arrangements are decreasing. The Dutch government has put the Social Support Act (in Dutch: Wmo) in effect, which decentralized the responsibilities for care and support to municipalities. This implies that municipalities, in cooperation with, for example, welfare organizations and community health services, are expected to support their citizens and provide support and assistance when needed, such as arranging domestic care, facilitating mobility aids, or house adaptations [6]. The Social Support Act stipulates that independent living is stimulated for individual citizens. In case of care or support needs, one needs to turn to one’s informal network before turning to formal care [7]. Hence, aging well and remaining in good health is essential for the aging citizen, and preparation for the changes and challenges that come with older age is an important issue. Given the enormous increase in the aging population and the related challenges, collective prevention for healthy older people, even before support and care needs arise, is desired.

Being well prepared for later life could benefit the prevention or delay of the onset of frailty. Age-related preparations comprise all the thoughts and activities related to adapting to age-related changes, intended to maximize positive outcomes, i.e., to ensure that one can age well [8–10]. Age-related preparation is a multifaceted, broad concept, which can comprise several types of preparation. For example, it can include *thoughts and awareness* about age-related changes; *anticipation and active preparation* regarding age-related changes; and *gathering information or discussing* age-related topics with others [8, 9]. Topics to prepare for can be anything ranging from financial issues, retirement, physical and mental health, or possible future care needs to looks and appearance, maintaining social relations, and arranging end-of-life related matters [8, 9]. Specifically, several aspects of age-related preparations are shown to positively impact (the progression of) frailty, such as developing or maintaining social relations [11], arranging home modifications [12], or having a healthy and active lifestyle [13]. Vice versa, aspects related to bodily and household disorder, particularly dress and hygiene, are associated with higher levels of frailty [14]. Furthermore, interventions that have been shown to reduce or prevent frailty have been aimed at, for example, exercise, nutrition, cognition, and psychosocial aspects [15].

Although age-related preparation could benefit frailty prevention and aging well for all aging citizens, research often showed that engaging in age-related preparations depends on individual characteristics and personal circumstances [16]. Barriers to preparing for older age include being of younger age [10], having a fatalistic outlook on health, or perceiving health and social care services as inadequate [17]. Stimulators for age-related preparation are perceiving age-related preparations as useful, perceiving fewer years left in life and having experience with caregiving and care needs in their close environment [16, 18].

Still, it are not only the aging citizens who have a responsibility in aging well. The municipalities, welfare organizations, and community health services have a significant role in the Social Support Act [19]. Given the immense increase in the aging population with its related challenges in the near future, these stakeholders need to understand how (future) older adults can be supported in old-age preparations in their local context. In previous studies, aging preparation has been assessed often at the individual level [10, 16–18]. However, the local context should be incorporated as well, since motivations, needs and preferences to prepare for older age differ so much among people. With this knowledge, middle-aged and older adults can be supported and stimulated to prepare for older age in a way suited to their circumstances, possibly preventing or delaying the onset of frailty and increasing or maintaining quality of life. In the current study, we aimed to describe and understand middle-aged and older adults’ focus points, needs, and wishes in preparation for older age. In doing so, we add to the understanding of how municipalities, welfare organizations and community health centers can support people in aging preparation in an urbanized context. Therefore, the research question is as follows: what are the focus points, needs and wishes of middle-aged and older adults regarding preparation for older age?

## Methods

The current study is a mixed-methods study undertaken with a convergent approach [20]. The study was conducted in a neighborhood called Oosterpark in Groningen, a city in northern Netherlands. The Oosterpark neighborhood has just over 12.000 inhabitants [21] and is one of the old neighborhoods in Groningen. Traditionally it is a working-class neighborhood, and in general, circumstances in this neighborhood are less favorable [22]. From 2023 up to 2042, the share of older adults in the Oosterpark neighborhood is expected to rise drastically: the age group 65–74 is expected to increase from 750 to 1300 people, while the group aged ≥ 75 years, currently 393, is expected to rise to approximately 1600 inhabitants [21]. From eight older neighborhoods in the city of Groningen, only the Oosterpark expects such a drastic rise in the 65–74 age group, and besides the Oosterpark, only one other neighborhood expects a similar strong rise in the ≥ 75 age group [21].

### Study design

**Fig. 1:**
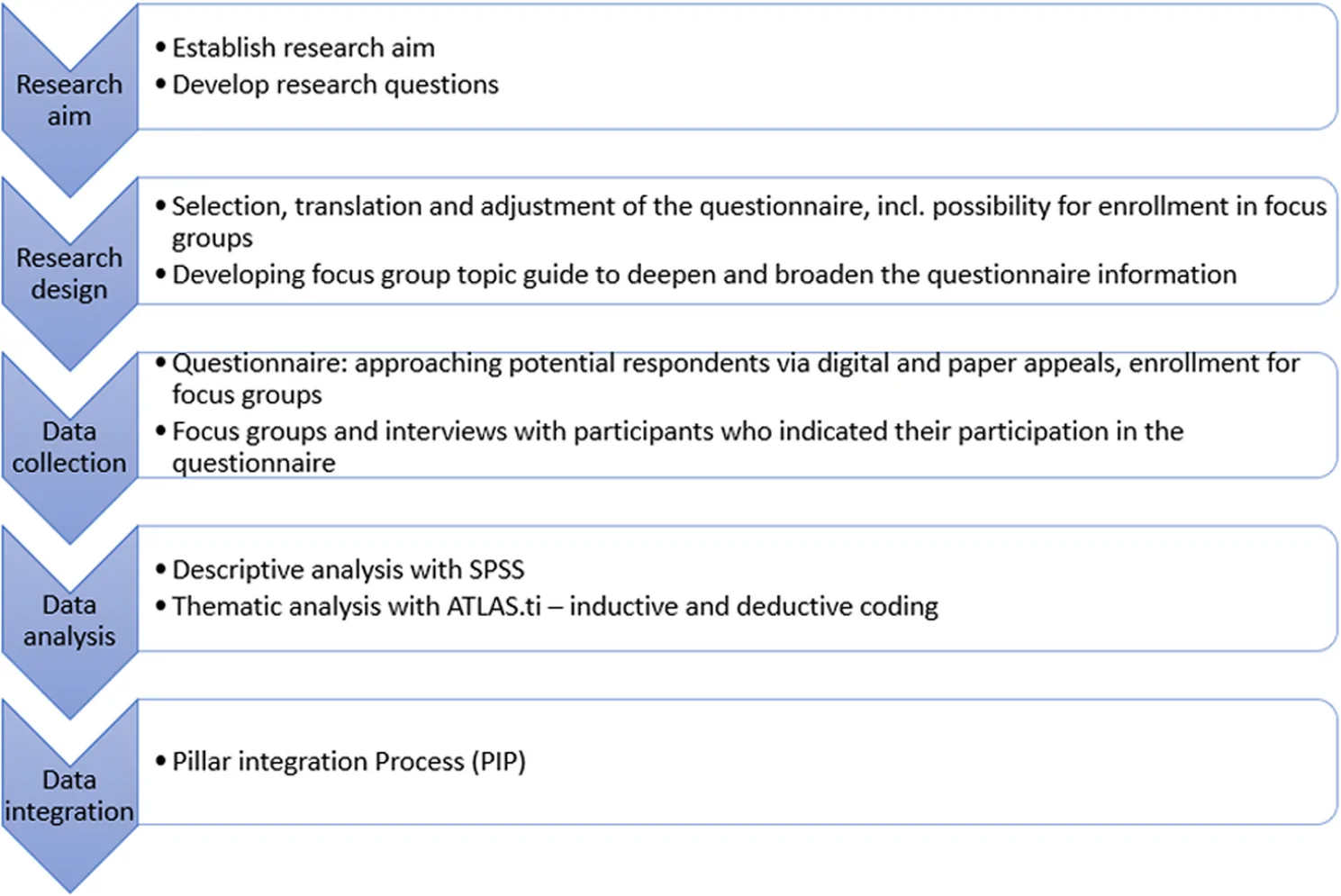
Procedural diagram of the study’s workflow

The purpose of the mixed-method design was to allow both data collection methods to elaborate, illustrate, and enhance each other and expand the data collection’s breadth [23]. We applied a convergent approach [20] in which qualitative and quantitative data was collected and analyzed simultaneously. Thereafter, the data was integrated and compared. The study’s workflow, including research design, data collection, data analysis, and data integration, is presented and explained in more detail in the procedural diagram in Fig. 1 below.

### Study population

The inclusion criteria for participants were: (1) Currently living in the Oosterpark neighborhood; (2) Currently being aged ≥ 45 years, hereby including middle-aged and older adults; and (3) Being able to understand and speak Dutch. Hence, Oosterpark inhabitants of younger age were excluded from the study. Participants were invited to take part in the study by announcements in local newsletters, in a door-to-door newspaper, and on social media channels from the local welfare organization WIJ. Furthermore, flyers were spread in the neighborhood, both door-to-door and in public places such as the community center, the local welfare organization, and neighborhood restaurants.

### Data collection

**Fig. 2:**
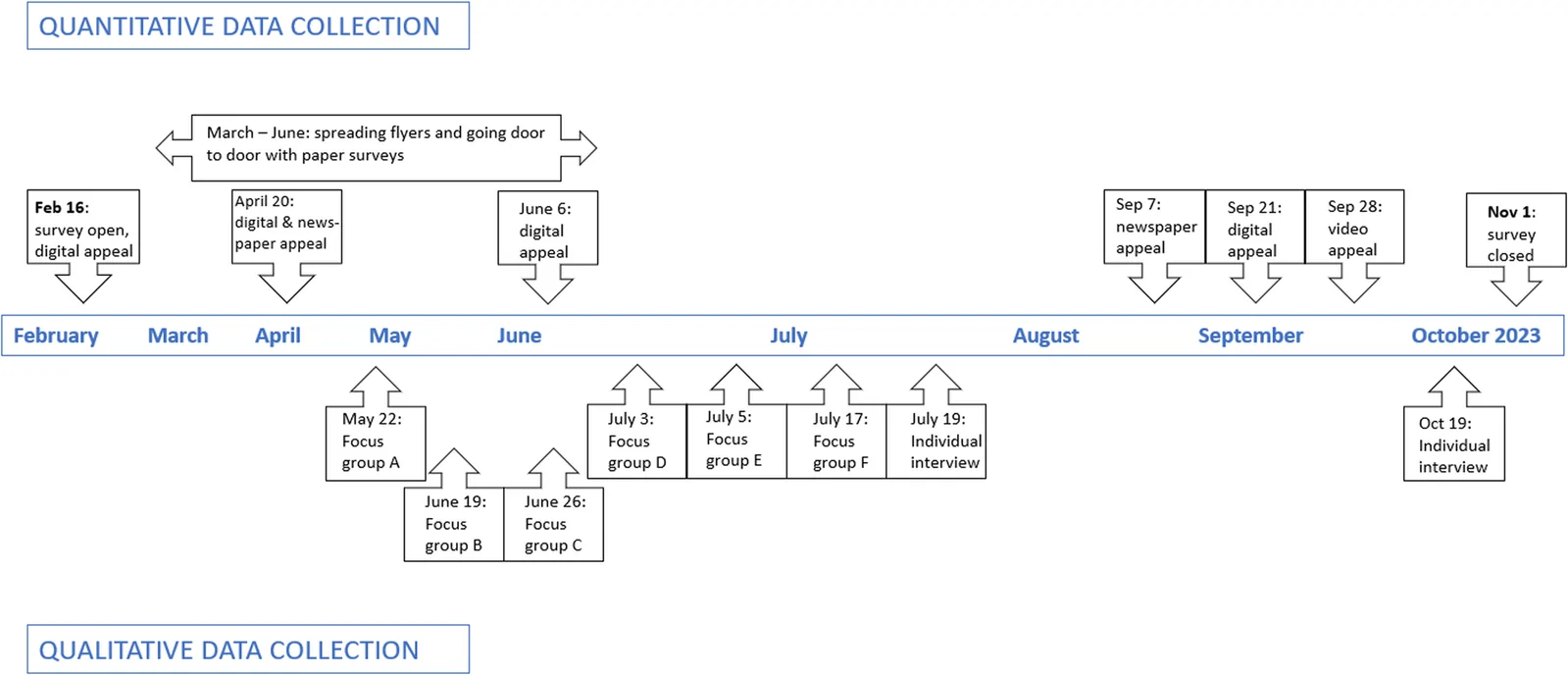
Timeline of the data collection process, ranging from February 16 to November 1, 2023

Both quantitative and qualitative data were collected to get a thorough understanding of age-related preparedness in the Oosterpark neighborhood [24]. Quantitative data were collected using a questionnaire that was developed to collect information on an extensive range of age-related preparations [9]. More specifically, the questionnaire included nine themes regarding age-related preparation, i.e., financial situation, emergency situation, mental and physical fitness, housing, looks and appearance, personal relationships and social contacts, health, leisure activities and commitments, work and professional activities. For each of the nine themes, three types of preparation were inquired about: if respondents were taking active preparations, if they thought about the theme, and if they collected information or discussed the theme with others. Participants gave their responses to each item on a 4- point scale with the options “not at all”, “a little”, “quite a bit”, and “a lot”. The questionnaire of Kornadt and Rothermund was developed in 2014 and since then used in other studies (e.g. [25]). It was translated in different languages and used in different cultural settings. For the current study, the English version of the questionnaire was translated into Dutch by a researcher and a native English speaker. Two researchers (FL and NK) compared and discussed the translated versions and reconciliated the two translations into one final Dutch version of the questionnaire, and assessed by a staff member of the local welfare organization WIJ Oosterpark. The final questionnaire was made available online in February 2023, accompanied by an appeal in the digital neighborhood newsletter. Starting in April 2023, questionnaires were collected by the first researcher (NK) and a research assistant by going door-to-door in the Oosterpark neighborhood. Some streets were mentioned specifically by the welfare organization WIJ, others were chosen randomly. Data collection was performed on varying days and times (e.g. mornings, afternoons and evenings), to increase the breadth of respondents. Several appeals were placed in the local door-to-door newspaper and digital neighborhood newsletter throughout the data collection period. In Fig. 2, an overview of the data collection period is presented.

In the questionnaire, respondents could indicate their willingness to participate in a focus group discussion. The choice to conduct focus group discussions as the qualitative data collection method stemmed from the idea that sharing and comparing experiences and perceptions between the participants would yield rich and extensive information [26].

Those respondents who chose to participate in focus group discussions were contacted by the leading researcher by email or telephone to schedule the appointment. The leading researcher conducted a total of six focus groups, and a research assistant was present during the first three sessions. For each focus group discussion, different participants were present, with a total of 23 participants. The focus group discussions were conducted in Dutch and took place in the community center in the Oosterpark neighborhood. This location was selected because it is in a central place in the neighborhood, a neutral place as opposed to the welfare organization, and accessible for people in wheelchairs and with wheeled walkers. Four to six participants were invited for each focus group discussion. Participants were selected for the first three focus groups based on similar age. In later focus group discussions, ages varied more. Finally, two individual interviews were conducted. The interviews were held with participants who were willing to participate in a focus group discussion, but were unfortunately unable to attend due to incompatible schedules. These interviews were conducted at the participant’s homes and followed the same procedures as the focus group interviews. Both the focus group discussions and individual interviews were audio recorded. The duration of the group discussions and individual interviews varied between 47 min and 1 h 51 min.

The topic guide for the focus groups and individual interviews was developed based on concepts and themes from age-related preparation research [9], and guided by the research aim. The research team and two staff members of the local welfare organization assessed the topic guide. After the first focus group discussion, minor additions were made to the topic list, such as the phrasing of a question and the order of two questions.

### Data analysis and integration

Quantitative and qualitative data sets were analyzed using advanced integration analytics, in which the data types are closely linked and interpreted jointly [24]. More specifically, the data analysis process proceeded using the following steps. First, the quantitative data were analyzed using descriptive and comparative statistics in SPSS. The data was divided into three age groups (≤ 54, 55–64, ≥ 65 years). Mean scores were calculated per questionnaire item, resulting in mean scores per preparation type and age group for each of the nine themes. To assess differences between age groups, ANOVA tests for differences in mean scores were conducted (α = 0.05) using SPSS, including only cases without missing data.

Second, the qualitative data were analyzed in ATLAS.ti using thematic analysis [27]. Codes were developed both inductively and deductively based on the questionnaire themes. A first coding scheme was drafted based on the themes of the questionnaire and the types of preparation. Throughout the coding process, in particular in the first transcripts, codes were added based on the issues raised by the participants. Coding was conducted by the first researcher (NK) in close consultation with the second researcher (FL): NK coded two documents, after which thorough discussion with FL took place. NK made adjustments to the coding scheme and coded the other transcripts. Throughout the remaining coding process, there was regular consultation and discussion with FL. In case of different interpretation or doubt, the complete research team was consulted.

Third, both data types were integrated based on the Pillar Integration Process (PIP) [28].

**Fig. 3:**
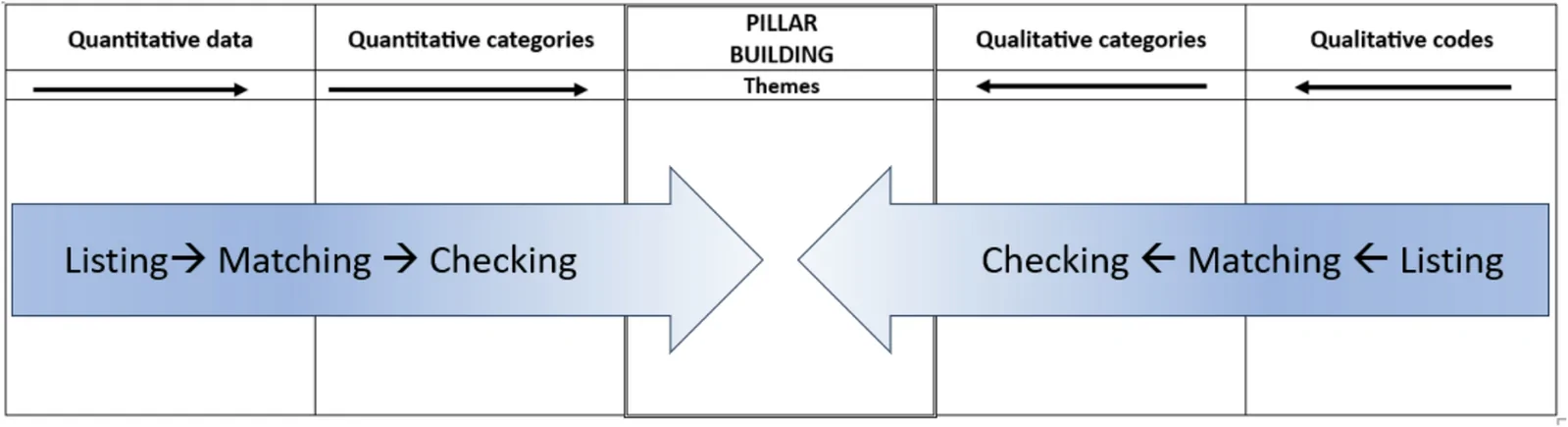
Representation of the Pillar Integration Process, based on Johnson et al., 2017

The Pillar Integration Process (PIP) is a data integration technique that entails listing, matching, and checking both data types, leading to pillar building, in which both types of findings are synthesized [28]. Integrating the data following this process enhances the added value of incorporating two different types of data in research. In Fig. 3, a visual representation of the PIP is presented based on the work of Johnson and colleagues (2017). NK proposed this data integration technique to the research team, upon which it was thoroughly discussed and finally agreed upon. The PIP was carried out by NK, in close consultation with FL. For each theme included in the questionnaire, a pillar integration table was composed, of which an example can be found in Appendix 1. To carry out the PIP, the following steps were taken. First, the quantitative data relevant to the theme at hand, was listed in the outer left column. Second, the matching categories or topics were extracted from this list and described in the second left column. Third, the qualitative data relevant to the theme was listed in the outer right column. Fourth, the matching qualitative categories or topics were extracted from this list and were described in the second right column. Finally, both the qualitative and quantitative findings were thoroughly compared and integrated in the middle column. Similarities and discrepancies were revealed during this process. Throughout the pillar integration process, findings and implications of the PIP were repeatedly discussed by NK and HR, and by NK and FL. Particularly, attention was given to conflicting results or discrepancies by discussing possible explanations. If deemed necessary, the relevant sections in transcripts and dataset were double-checked. The complete research team was consulted periodically for advice.

### Reflexivity

The two primary researchers are both experienced in aging research, and have a background in social sciences and public health (FL). The first researcher followed interview training before, and in addition, prior to this study, received thorough instructions and practical tips from an experienced researcher regarding focus group discussions. This aided in improving interview techniques and awareness of possible prejudices.

During the focus group discussions and individual interviews, time was taken for a personal welcome and building rapport with the participants. Furthermore, the topic guide contained open-ended questions.

### Ethical approval

Ethical approval for the study was granted in December 2022 by the Ethical Advisory Committee of the Hanze University of Applied Sciences (approval number heac.2022.033). Questionnaire respondents received information about the research digitally or orally and were offered an information letter about the research purpose, design, and procedures. The respondents provided informed consent and were allowed to ask questions before, during, or after completion of the questionnaire. Focus group participants received an information letter via email or regular mail, including the research purpose, design, and procedures. They were allowed to ask questions before, during, or after the focus group sessions. Following the focus group session, focus group participants received an interview abstract to check for any inaccuracies or to provide additional information. This yielded two additional remarks: respondents extended their argument on a specific topic in both cases.

## Results

The findings of the data analysis are presented below, starting with the quantitative results, followed by the qualitative results, and ending with the integration of both data strands.

### Quantitative results

The age-related preparedness questionnaire was (partly) filled out by 205 respondents. With around 3600 inhabitants aged ≥ 45 years in the Oosterpark neighborhood, this amounts to a response percentage of 5,7%. For the analysis, we used only the completed questionnaires (*n* = 134, response percentage of 3,75%). The mean age of the respondents was 59 years (SD = 12). More characteristics are presented in Table 1 below.

**Table 1:** Socio-demographic characteristics of the questionnaire respondents

Socio-demographic characteristics	Categories	*N*
Sex	Female	91
	Male	37
	Different	2
	Do not want to answer	4
Marital status	Married or living together	45
	Single	56
	Divorced	20
	Widowed	13
Education level	No or primary education	4
	Secondary education or vocational education	41
	Higher vocational education or university	89
Monthly net household income	€600,- or less	1
	€601,- to €900,-	1
	€901,- to €1200,-	20
	€1201,- to €1500,-	15
	€1501,- to €1800,-	12
	€1801,- to €2100,-	8
	€2101,- or more	52
	I do not know/ I do not want to answer	25
Age in categories	≤ 54 years	44
	55–64 years	42
	≥ 65 years	48

**Fig. 4:**
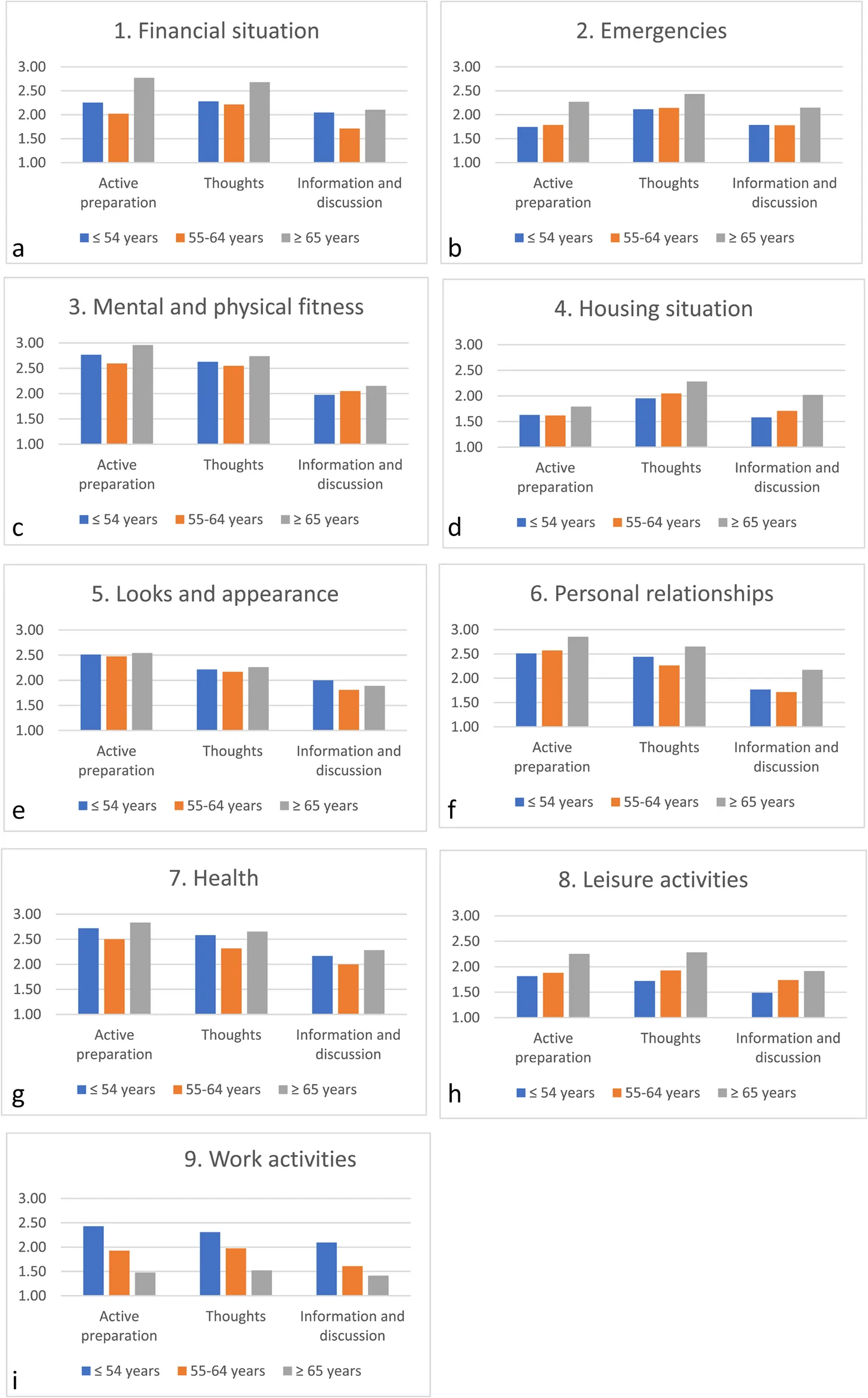
**a** Mean scores for *financial situation* per preparation type and age group. **b** Mean scores for *emergencies* per preparation type and age group. **c** Mean scores for *mental and physical fitness* per preparation type and age group. **d** Mean scores for *housing situation* per preparation type and age group. **e** Mean scores for *looks and appearance *per preparation type and age group. **f** Mean scores for *personal relationships* per preparation type and age group. **g** Mean scores for *health* per preparation type and age group. **h** Mean scores for *leisure activities* per preparation type and age group. **i** Mean scores for *work activities* per preparation type and age group

The quantitative data yielded insight into the respondents’ focus points regarding age-related preparations. Overall, the data revealed considerable differences between the themes and the nature of preparations. Considering the themes, results showed that *mental and physical fitness* gained the most attention by the respondents, followed by *health*. *Emergencies*,* housing situation*, *leisure activities* and *work activities*, on the other hand, received much lower scores. In general, regarding age groups, the ≥ 65 years age group had higher preparation scores for the different themes. Exceptions were the themes of *work and professional activities*, for which the ≥ 65 years age group had the lowest mean scores, and the youngest age group had the highest mean scores. Also, for *mental and physical fitness*, there were no significant differences in scores between age groups. Furthermore, considering preparation type, it became apparent that gathering information or discussing a theme with others, consistently received lower scores than the other preparation types. Active preparation received the highest scores for most themes, although for *emergencies* and *housing*, thinking about the theme was done the most. Mean scores for the themes are presented in Fig. 4a until i, per preparation type and age group. Response options ranged from 1 (not at all) to 4 (a lot).

*Financial situation* was considered most by the ≥ 65 age group. Contrastingly, the 55–64 years age group scored lowest on all types of financial preparation. In general, mean scores were relatively low for *emergencies*. The ≥ 65 years age group significantly showed the most attention to emergencies.

*Mental and physical fitness* was a theme in which a large majority of the respondents actively engaged, regardless of age group. Scores were lower for the other preparation types. Related to *housing situation*, respondents of all ages scored relatively low for all three types of preparation. Thinking about housing situation received the highest mean scores compared to the other types of preparation.

The quantitative data revealed similar scores for all age groups regarding *looks and appearance*. Considering *personal relationships and social contacts*, the ≥ 65 years age group scored statistically significantly higher than younger age groups on gathering information or discussing the theme.

Regarding *health*, active preparation received the highest mean scores for all age groups. Findings revealed that for *leisure activities*, older respondents (≥ 65 years) consistently considered all types of preparation significantly more than the ≤ 54 years age group. Regarding *work situation*, the ≤ 54 years age group consequently and significantly displayed the highest mean scores for all preparation types.

### Qualitative results

**Fig. 5:**
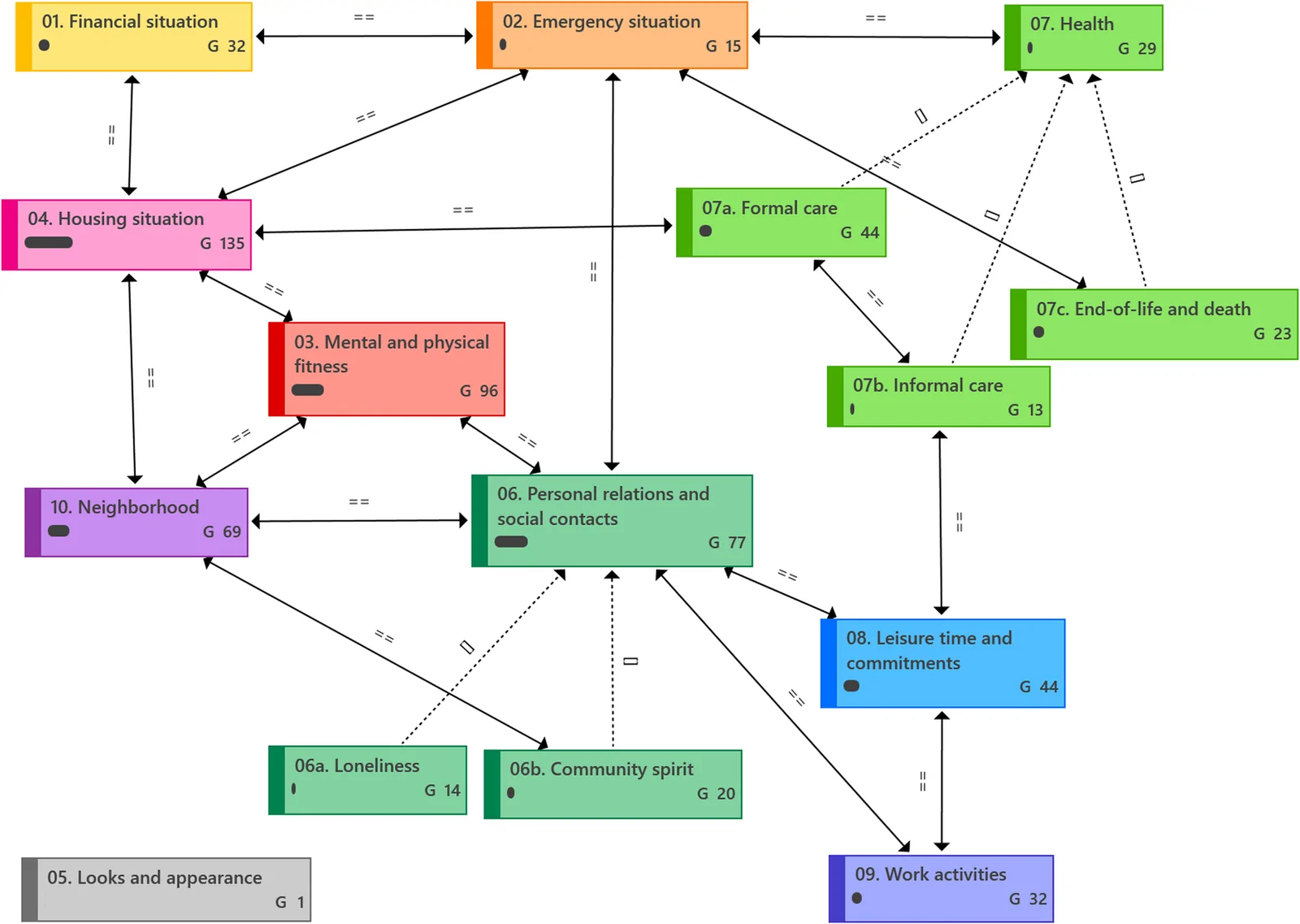
Themes, subthemes and their relations as identified by the participants. Solid lines (accompanied by ==) indicate associations between themes, dashed lines (accompanied by [] ) indicate subthemes. The G indicates frequency of themes, e.g., personal relationships was discussed 77 times, looks and appearance once

Six focus group discussions were organized, in which 23 participants participated. Group sizes varied from two to six participants. In the end, two participants were interviewed separately, because of incompatible schedules. The qualitative data provided insights into the participants’ focus points, needs, and wishes regarding age-related preparations. Also, the participants identified additional (sub)themes. Specifically, a theme on the living environment was mentioned (outside of the household situation), which we conceptualized as a new theme called *neighborhood.* This theme was considered by many participants and connected to a range of other themes, such as *personal relations*, *housing* and *mental and physical fitness*. Within existing themes, some smaller issues were identified, which we label as ‘subthemes’. For the theme *health*, participants identified the subthemes *formal care*,* informal care*, and *death and end-of-life.* Within the theme *personal relationships and social contacts*, they described the subthemes *loneliness* and *community spirit*. Furthermore, participants reported to perceive associations between certain themes. In Fig. 5, associations between existing questionnaire themes as well as the newly identified (sub)themes are presented.

#### Theme 1: Financial situation

Participants experienced much uncertainty about their financial situation in relation to aging and had many questions about this theme. Major topics were, for example, income after retirement, income when a partner dies, life insurance, changes in pension funds, and long-term financial planning. However, participants indicated they did not actively prepare or gather information about this theme.

Participants mentioned that financial support from the government was desired in specific situations: for older adults with care needs who lack an informal network, for exercise and recreation for older adults without financial capacity, and as compensation for work-related activities after retirement.

#### Theme 2: Emergencies

Within the theme of emergencies, participants identified four topics: (1) End-of-life and death, i.e., arrangement of a (living) will, specific wishes related to the last phase of life, and practical arrangements (e.g., how to arrange a funeral). One interview participant was working on a binder including all the practicalities surrounding end-of-life: *“An emergency binder. If I end up in a crash*,* for example*,* in the binder is described what should be arranged*,* where and how to find it. [Having] a certain way to organize these things is a good idea.”* (Female participant, age group ≤ 54) ; (2) Support and help in an emergency or sudden event, i.e., where can one turn for help and support, which facilities exist, and how can one use them or qualify for them. This was also related to the particular or constructional limitations of the home; (3) Financial situation in case of emergency: how does sudden death impact the financial situation of the remaining family, how should insurance be arranged; (4) Informal network, i.e. who will be there for you to provide support and care in case of emergency situations?

#### Theme 3: Mental and physical fitness

Participants considered mental and physical fitness essential in aging preparedness, but differed in their preferences related to sports facilities and activities. Some actively engaged in physical exercise, e.g., going to the gym, or working out at home. Others indicated remaining active through activities such as walking, climbing the stairs, or cycling. Furthermore, we found contrasting preferences such as exercising alone versus exercising with others; exercising with people from similar ages versus exercising with people from all age groups.

Some participants perceived a responsibility to remain resilient and to have the ability to adjust to aging: *“I am quite fit*,* but suddenly I have a knee injury and my hips hurt (…) You need to go on with everything*,* but you constantly need to adjust to what [is possible]”* (Female participant, age group 55–64). Others proposed a preventive approach by volunteers going door to door for an annual check on senior citizens.

#### Theme 4: Housing situation

Participants thought about their housing situation quite a lot, and several issues arose concerning the type of housing available for seniors. First, participants expressed interest in collective living for seniors, combining shared and private space. However, they were pessimistic about the feasibility of such type of housing in the Oosterpark neighborhood. Second, senior friendly apartments or homes for independent living, adapted for older age, were desired in the neighborhood. According to participants, the government should develop more senior-friendly homes, possibly in cooperation with home care organizations. Third, the possibility to combine home and care, i.e., the idea of a care home within the neighborhood was a strong wish among participants. Prioritizing Oosterpark inhabitants in admittance to such a facility was considered essential. Next to the type of housing, the house interior should be suited for old age, and it should be possible to make adaptations for older age. The housing corporation was considered a major player, e.g. by installing a stairlift or an additional toilet on the first floor. In addition, some participants were occupied with safety of the house by conducting repairs and getting the house ready for the future.

Housing was also related to other topics. Concerns were expressed that when one had to leave the neighborhood, one would also lose their social network of neighbors and friends: *“Where I live*,* the renting neighbors have good relationships. (.) If someone needs groceries*,* we help each other. But if you become less mobile*,* you’ll have to move houses because of all the stairs.”* (Female participant, age group ≤ 55). Furthermore, the physical environment of the neighborhood and available facilities such as supermarket, GP, and other health professionals were important. Finally, one participant expressed the idea of guidance for this later stage of life, including advice about living situations and possibly about moving houses.

#### Theme 5: Looks and appearance

Although looks and appearance were one of the nine themes in the questionnaire, they were not mentioned at all during the focus group discussions and individual interviews.

#### Theme 6: Personal relationships and social contacts

A large share of participants mentioned actively preparing for older age in relation to social contacts. Many participants gave examples of how they remain socially active. Within the existing theme of personal relationships and social contacts, participants identified two important subthemes, namely *loneliness* and *community spirit*.

- *6a. Loneliness.* Participants expressed concerns about older adults in the Oosterpark neighborhood living alone and lacking a social network. In this respect, participants also mentioned the importance of the possibility of meeting others in the neighborhood, which they felt was insufficient. One individual noted that she feels lonely sometimes despite having a partner and having casual contact with neighbors. She partly explained this loneliness by losing contacts and a sense of belonging after retiring from her job. This was recognized in several focus group discussions: social contacts can be lost due to retirement, and your network might become smaller.
- *6b. Community spirit.* Participants experienced a lack of community spirit. Individualism was mentioned as more common nowadays, with community spirit and ‘being there for each other’ as something from the past. However, it was felt as particularly important in aging. As a female focus group participant explained: *“[Being asked] if you need groceries, or you offer someone to drive them to the hospital. I consider it very important, when you get older. One can do this, the other can do that, at together you’ll manage.”* (Female participant, age group 55–64).

#### Theme 7: Health in old age

Participants gave much consideration to health, related to specific concerns. Within the existing health theme, three subthemes were identified: *formal/professional care*, *informal care*, and *death and end-of-life*.

- *7a. Formal/professional care.* Much unclarity and several worries related to formal and home care were identified. For instance, participants were concerned if facilities such as a general practitioner or physical therapist would be available in the neighborhood. Participants expressed the need for less complex ways to arrange care and support: many experienced unclarities regarding the application for care and support. Furthermore, concerns were expressed about whether home care will be sufficient in the coming years. Besides, participants indicated that care for older adults with specific needs would be desired in the neigborhood. A participant stated: *“And that is also part of aging: care needs to be arranged well. Also for older people with mental issues. And I also have issues*,* I have many issues. And I probably will be a difficult person when I am older. But which facilities are available in our neighborhood*,* for fhose people?”* (Female participant, age group ≤ 54).
- *7b. Informal care.* Informal care received much consideration related to aging preparedness. Participants were aware of the need to call on their informal network in case of needs. Some participants explained they were presently occupied with informal care for parents or a partner, of have been in the past. Others did not have experience with informal care yet but questioned how they would deal with it themselves, given the burden it can pose on informal carers. For example, a participant explained that he hesitated whether he could or would want to provide informal care to his partner, if it would be needed.
- *7c. Death and end-of-life.* The health theme was often related to wishes and practicalities regarding end-of-life and death. A majority of participants indicated to need help drawing up a will or a living will. Furthermore, they expressed the need for support surrounding end-of-life. When one wants to terminate one’s life, this should be made possible more easily with professional help and support. A participant emphasized the need for attention for this topic and the importance of breaking the taboo surrounding death and end-of-life.

#### Theme 8: Leisure activities and commitments

Findings revealed several discrepancies between the age groups concerning leisure activities. The younger participants desired organized activities, not specifically for older adults but for all ages. Specifically, they desired fun and diverse activities, with high attendance. On the other hand, older participants did not express a need for organized activities but took the initiative to remain active and join existing activities. In addition, the data revealed that older participants have spent time in finding suitable leisure activities. They thoroughly considered their motivations to engage in certain activities or commitments. Related to this, the older age groups valued the freedom to use and divide their time, balancing commitments and leisure.

#### Theme 9: Professional activities in old age

Participants focused on the change from work to retirement and how to prepare for a new phase in life. Especially the participants that were not retired yet, had many questions and thoughts about preparing for older age concerning their work situation. For example, they wondered how they could prepare for the transition from working life to retirement. Participants expressed uncertainties and concern about which changes they would face during this process, e.g., about losing social contacts and how to preserve meaningful connections with others. In addition, participants lacked information about working despite older age.

Regarding activities after retirement, one of the participants proposed the idea of financial rewards for older adults who still participate in valuable activities in the community or neighborhood. The financial compensation could acknowledge the importance of people’s participation and perpetuate the commitment.

#### Additional theme 10: Neighborhood

The neighborhood emerged from the focus group discussions as a major theme: participants emphasized that familiar surroundings are essential in aging and that knowing the neighborhood is beneficial. Participants shared their active involvement in the neighborhood by keeping the streets tidy and clean and reporting defects to the municipality.

Furthermore, participants considered the availability of facilities in the Oosterpark neighborhood essential, e.g., housing, meal services, places to meet others, possibilities for exercise, and cultural facilities. “*If you move*,* you need to rebuild a social network*,* which is difficult at this age. You are less mobile*,* so it is hard to go somewhere. (…) I feel at home here*,* I know how things work here*,* [I know] the neighborhood*,* the environment. I think most older people will feel like that*.” (Male participant, ≥ 65 age group). Also, the community center was cited as very important, and participants expressed their hope for it to remain active in the years ahead.

Regarding the physical environment, concerns were expressed, such as sidewalks filled with bicycles or scooters and loose tiles, particularly for people with walking aids: *“Loose tiles on the sidewalks matter nowadays. [When I was young] loose tiles were there as well. But it did not matter*,* your body just adapted. But now I have to be careful*,* with everything.”* (Male participant, ≥ 65 age group). Other problems concerned pedestrian crossings, considered unsafe, and a lack of (functioning) streetlights, which present difficulties for people with slower gait or declining vision. According to the participants, these circumstances restrict older adults from going out alone. Furthermore, for older adults, facilities such as supermarkets were perceived to be beyond walking distance. Finally, daytime facilities in the neighborhood for adults with, for example, memory issues were missed but considered necessary.

### Integrated findings

The quantitative and qualitative data were synthesized following the Pillar integration process (PIP). To exemplify this approach, the Pillar integration model for theme *emergencies* can be found in Appendix 1. Findings and related implications revealed in the PIP are described in Table 2 below.

**Table 2:** Findings and related implications per theme, extracted from the Pillar integration process

Theme	PIP findings	Possible explanations
1. Financial situation	-Older participants and respondents consider this theme more than younger age groups.	-Day-to-day worries, situations and problems might prohibit people from preparing for their future financial situation.
	-While much uncertainty regarding future financial situation was expressed, engagement in preparation was relatively low.	
	-The role of the government for financial support is expressed by participants and respondents.	-A governmental responsibility is perceived in financial support.
2. Emergencies	-Several topics related to emergencies received attention, mainly regarding a lack of knowledge: end-of-life and death, and support and help in case of emergency were identified both in the quantitative and qualitative data.	-A knowledge gap seems to be identified regarding practical solutions in case of emergencies.
	-Few people seemed actively engaged in this topic, regardless of age.	-Preparation for emergencies seems largely dependent on personal experience.
3. Mental and physical fitness	- Many participants and respondents actively engaged in preparation for this theme.	- Mental and physical fitness was a matter of active involvement, and less a matter of ‘theoretical’ preparation.
	-A share of the participants and respondents perceived own responsibility and initiative in remaining fit, while others expressed reliance on other parties, e.g. regarding Social Support Act or organized activities.	-Concerns with the changes in the Social Support Act in the years to come, indicate an (expected) dependency on support.
4. Housing situation	-Housing received low scores in the quantitative results but emerged as an essential concern in qualitative data.	-The PIP revealed discrepancies between the quantitative and qualitative data.
	-Worries were expressed about the availability of suitable housing and the possibility of home adaptations or moving houses within the Oosterpark neighborhood.	-The role of external parties seemed to be crucial. Participants and respondents experienced a major dependence on external players (e.g. municipality and housing corporations), while they lacked a sense of being in charge of their situation. This might prevent individual action preparation aimed at housing.
	-Collective living gained much attention in both qualitative data and open questions in the questionnaire.	
	-Housing was related to other important topics such as care, social relationships and neighborhood.	
5. Looks and appearance	-Although looks and appearance were one of the nine themes in the questionnaire, they were not mentioned during the focus group discussions and individual interviews.	-These findings might indicate that people do not feel comfortable discussing the topic in a group setting.
	-In the questionnaires, gathering information and discussing the topic with others received lowest mean scores compared to active preparation and thoughts about the theme.	-Another explanation could be that participants do not relate looks and appearance to aging preparation.
6. Personal relationships and social contacts	-Integrated findings confirmed that a large share of participants and respondents indicated to be actively preparing for older age in relation to social contacts.	-The association of loneliness with retirement indicates that different phases in life can impact social relations. This effect might even be enhanced by the low level of community spirit that was identified in the data: if existing social relations diminish, and if people spend more time in their home environment due to retirement, and in addition, community spirit is also perceived as low, feelings of loneliness could further increase.
	*-Loneliness* and *community spirit* were subthemes recognized in both the qualitative data as well as the open questions in the questionnaire.	
7. Health in old age	-Although active preparation for health received the highest mean scores in the questionnaires, participants in the focus groups spend much thoughts on health, and they expressed several concerns.	-For the identified subthemes, the emphasis was on an information gap: how to arrange care, how to arrange practicalities or support surrounding death and end-of-life? Also the moral side of informal care was considered: are people able and willing to provide or receive informal care?
	-Within the existing health theme, participants and respondents identified three subthemes: *formal/professional care*, *informal care*, and *death and end-of-life*.	
	Participants and respondents identified three different roles in health preparation. The first was preparation in the personal atmosphere and everything individuals can do to remain healthy or adjust to one’s health situation. Second, there was the level of professional care, including the concerns regarding care supply in the Oosterpark neighborhood, and home care facilities. Third, participants recognized the role of the government and municipalities, and policies such as the Social Support Act.	-These findings implicate a discrepancy between proactive preparation for the future and maintaining self-direction versus reactive behavior and dependence on care and support.
8. Leisure activities and commitments	-Although older participants and respondents (≥ 65) considered leisure activities more important than younger age groups, the younger age groups had clear opinions and ideas about leisure activities.	-The main discrepancy between younger and older age groups seemed to be the desire for organized activities by external parties, versus finding suitable leisure activities by themselves.
9. Professional activities in old age	-In the questionnaire, this theme was aimed at activities regarding adjustment in work and professional activities, such as changing to less physical work or following courses to take on different tasks. In the focus group discussions and interviews, the participants focused instead on the change from work to retirement and how to prepare for a new phase in life.	-Results imply a different perspective on the theme. This difference might also be explained by the uncertainty and lack of information that was perceived by participants who were not retired yet.
Additional theme: 10. Neighborhood	-The neighborhood was not included in the questionnaire but was an important theme in the qualitative data. Specifically, familiarity with the surroundings were considered crucial in the aging process.	-Neighborhood is a crucial aspect in aging and related to a range of other topics, according to participants.
	-Neighborhood facilities and physical environment were considered important. These issues were related to housing, social networks, physical fitness and health.	
Additional finding: No preparation	-Many respondents and participants indicated that they have not prepared for older age (yet), for varying reasons: feeling too young and perceiving no reason yet to prepare for older age; not knowing how to prepare for later life; not knowing what they should or could prepare for.	In this vein, a low-key information point for older people was proposed, where they could ask questions related to a broad range of topics related to age-related preparations.

## Discussion

The current study aimed to describe and understand middle-aged and older adults’ focus points, needs, and wishes in preparation for older age. Themes considered in the study were financial situation, emergency situation, mental and physical fitness, housing, looks and appearance, personal relationships and social contacts, health, leisure activities and commitments, work and professional activities. In addition, participants identified the theme *neighborhood* and several subthemes, in which *formal care*, *informal care*, and *end-of-life and death* emerged as essential health issues and *loneliness* and *community spirit* were identified as part of personal relationships and social contacts. Several findings of the study clearly stand out. First, certain themes are much more likely to be considered than others by middle-aged and older adults. In particular, neighborhood gained much attention and was related to several other themes, such as housing, social relationships and facilities or services. In addition, although people give much thought to several themes, they lack active preparation. Finally, we found that many middle-aged and older adults do not prepare for older age. They may be unaware of the importance or do not know how to prepare, or to access support. It can also stem from a passive attitude, in part caused by a perceived dependency on other parties. This gap in knowledge and connected lack of action regarding age-related preparation is recognized by the participants of this study and is perceived as a concern by many.

### Focus points, needs and wishes in age-related preparation

This study exposed the importance of certain (sub)themes according to middle-aged and older adults. First, as mentioned above, *neighborhood* was distinguished as an additional theme, emphasizing the essential role of the local context in age-related preparedness. Active preparation concerning the neighborhood was aimed at day-to-day actions such as keeping the neighborhood clean and tidy, or reporting defects such as loose tiles, to the municipality.

However, middle-aged and older adults expressed many concerns and thoughts about the Oosterpark neighborhood. For example, the safety of pedestrian crossings, accessibility of sidewalks, or distance to facilities such as supermarkets, particularly related to physical mobility or visual impairment in older age. This aligns with previous research stating that when people experience safe passage and good access to services and facilities, the use of neighborhood space and engagement within the community is stimulated [29].

Our findings further indicated that middle-aged and older adults related *neighborhood* to themes such as *housing* and *personal relationships*. Concerns are expressed that senior-friendly housing is lacking in the Oosterpark neighborhood, but existing social contacts will be lost when people move to another neighborhood. This emphasizes the importance of familiar faces and personal relationships, and support the idea that neighborhood familiarity offers constancy in place and social relationships, which positively impacts the abilities of older adults [29]. These findings support the necessity of an integral approach towards the aging population, that takes the physical and social environment into account.

Second, people strongly relate *health* to *formal care*, *informal care*, and *end-of-life and death*. Many uncertainties and concerns arise relating to those themes. Middle-aged and older adults question if formal care will be fitting their specific needs and preferences, and if it will even be available for them in case they needed care. This aligns with research that found that a perceived inadequacy in formal care services could be a barrier to preparation [17]. In addition, much uncertainty and doubts in arranging for care and support exist among middle-aged and older adults. This knowledge gap might even enhance the inability to prepare for this theme. Moreover, many people feel that they cannot prepare for their future health because of the uncertainty of the future. Unforeseen circumstances may arise which one cannot control. This adds to a previous finding that a fatalistic perception of health could be a barrier to age-related preparation [17].

In addition, middle-aged and older adults who experienced situations such as providing informal care, close ones suffering from dementia, or the final life phase of a close one, often do consider their future health, care needs, and end-of-life. Having had these experiences makes them more aware of their future situation, which confirms previous findings [16, 18]. Remarkably, participants hardly connect these experiences to arranging possibilities for potential informal care for themselves.

Third, *personal relationships and social contacts* were related to *loneliness* and *community spirit*. Middle-aged and older adults desire more community spirit and less loneliness in the neighborhood. These are important findings, not only because of the high number of single households in the Oosterpark neighborhood, but also considering the body of literature on this subject. A recent systematic review found positive associations between social vulnerability (e.g., loneliness, low social support, low social participation) and frailty, indicating that higher levels of social vulnerability align with higher frailty [30]. Similarly, a Delphi study conducted in The Netherlands indicated that social support and informal networks in the neighborhood positively influence aging in place [31]. Moreover, engaging in social activities and maintaining social contacts is found to be helpful in preparing for the future and is considered crucial in healthy aging [32, 33]. Again, these findings emphasize the essential role of neighborhood context in aging preparation.

The study revealed the different focus points of middle-aged and older adults within age-related preparedness. During data collection, specific questions directly aimed at the different themes were purposely avoided, to understand with which themes the participants were occupied. While themes such as *mental and physical fitness*, *housing* or *care* were perceived as essential, the themes *emergencies* and *looks and appearance* were considered significantly less. *Looks and appearance* were not mentioned during the focus groups, and received low scores in the questionnaires. In contrast, themes such as *housing* or *mental and physical fitness* were considered extensively by participants. This leads us to believe that *looks and appearance* is not a much considered theme in aging preparation. Differences in aging preparedness between domains or themes are in line with previous research [16]. Possible explanations might be that people do not specifically relate looks and appearances to preparations for later life, or that they do not feel comfortable discussing the theme in a group setting. Considering the few people who seemed very engaged with emergencies, regardless of their age, preparation for emergencies might be largely dependent on one’s own experience or situation [16].

Furthermore, the type of preparation differed between the themes. For example, while many middle-aged and older adults actively prepare for *mental and physical fitness*, gathering information or thinking about this theme was reported to a lesser extent. On the contrary, people think and even worry about *housing* a lot, but hardly engage in active preparation. Instead, dependence on more prominent players such as municipalities and housing corporations is experienced. These findings align with previous research in which barriers to age-related preparation were found to exist when themes are perceived to be outside people’s influence [17, 34].

Finally, although the themes in the questionnaire are provided with explanations regarding content, people understood certain themes differently. For example, in the questionnaire, the theme *work and professional activities* is about adjustments in the work situation, e.g. by adjusting ones (physical) tasks. However, in the focus group discussions and interviews, the theme was discussed in light of the transition from work to retirement. The work-to-retirement transition is also a major topic in age-related preparation literature, e.g., in relation to retirement satisfaction or (un)paid activities after retirement [35, 36].

### Perspectives on age-related preparation

Older adults were more active in preparing for the future than younger adults. This supports previous research in which older adults of higher age are more inclined to prepare for the future than ‘younger’ older adults [37]. Also, a part of the middle-aged and older adults do not prepare for a range of different reasons. This is in line with findings from a qualitative study among Dutch older adults [38]. Furthermore, perspectives of middle-aged and older adults on their role in aging preparation differ. While some indicated that preparation is not always possible, or that the government or municipality needs to support older adults more, others emphasized the responsibility of individuals in preparing for older age. Among the people who emphasized this personal responsibility, we found the same duality between pragmatic and moral perspectives, that was recently found in Norway [4]. On the one hand, age-related preparations benefit the individual and society by maintaining healthy and independent older adults, who, in turn, are then better positioned to contribute to society. Considering the decline of the welfare state and shortages in health care, this might even be a necessity, which should be recognized and supported by policy. On the other hand, preparation for older age is perceived as an individual responsibility that mainly serves the interests of the individual and his or her social environment.

### Strengths and limitations

The current study has some strengths and limitations worth mentioning. A strength of the study is the mixed-methods approach, which provides additional insights and perspectives to the data. The integration of the quantitative and qualitative data adds depth to the interpretation of the participants on particular themes. Using the Pillar Integration Process not only reveals similarities, but also discrepancies between qualitative and quantitative results, thereby strengthening the findings. A second strength is the specific local approach to the study, taking into account the personal conditions and direct living environment of the middle-aged and older adults. Moreover, it is illustrative for similar neighborhoods where inhabitants feel attached, but are less suited for the aging population.

A limitation is the lower response rate of the study: we only reached around 5% of the 3600 inhabitants aged ≥ 45 years. Involving specific populations is a well-known issue in research, and the length or wordiness of the questionnaire might have played a role, when considering the difference between incomplete and complete questionnaires: apparently, many respondents quit prematurely. Furthermore, there might be potential self-selection bias: it could be possible that participants and respondents who took part in this study, were already interested in aging well or in issues related to themes as neighborhood or housing, for example. However, the wide range of opinions and experiences that emerged from the focus group discussions and the questionnaires, do not seem to imply self-selection bias.

A possible limitation regarding the qualitative data collection is the fact that two participants were not able to attend the focus group discussion, but were interviewed separately. Also, one focus group discussion had only two participants due to last-minute cancellation of one participant and a no-show of a second participant. This could have impacted the dynamic of the discussion. This was unfortunate, since we explicitly chose the method of focus group discussions so participants could share and compare experiences and ideas [26]. However, the procedure and topic guide used in the interviews and in the focus group with two participants were identical to the other focus groups. In the focus group discussion with two participants, discussion was stimulated by probes and follow-up questions by the researcher, similar to the other focus group discussions. Furthermore, during the process of coding and analyzing the data, there was close collaboration and consultation between the researchers. No remarkable differences were found compared to the other focus group discussions, and therefore we treated the data from the individual interviews and the two-person focus group discussion the same way as the data from the other focus group discussions.

### Implications for practice & research

The study has several implications for research and practice regarding age-related preparation. Middle-aged and older adults consider physical environment, social relationships, and facilities in the neighborhood essential in relation to aging-related preparedness. It was emphasized to such an extent that we propose it as an additional theme to the questionnaire. More in general, the importance of neighborhood implicates that research regarding age-related preparation should take into account the physical and social living environment of older adults.

A policy concern resulting from this study is the lack of action people display in age-related preparation. Although people spend much thoughts on certain themes, at the same time they report taking little or no action to prepare for the future. Considering the benefits age-related preparations can have regarding the onset and progression of frailty [11–13], it is critical for policy-makers to understand the reasons for these discrepancies, and how to address them. We observed that a lack of awareness and practical knowledge seem to underlie this issue. Middle-aged and older adults do not know which concrete actions they can take, or they are unaware of the possibilities for support or help. Furthermore, expected dependency on third parties hinders the feeling of being in control and prevents active preparation by individuals. Policy makers should acknowledge and support the changing role of citizens in preparing for older age, also given the declining welfare state and increasing shortages in health care. Providing a low-key information or service point where people can turn to for a broad range of questions could serve as a first step in the right direction.

## Conclusion

The mixed-methods study undertaken in a city district in northern Netherlands aimed to describe the focus points, needs and wishes of middle-aged and older adults, regarding age-related preparation. Middle-aged and older adults identified additional focus points: *neighborhood* emerged as a new theme; *formal care*, *informal care*, and *end-of-life and death* emerged as subthemes to *health*; and *loneliness* and *community spirit* were identified as subtheme to *personal relationships and social contacts*. Certain themes are much more likely to be considered than others by middle-aged and older adults. In addition, many people are concerned with age-related preparation, although little engage in active preparation. Finally, some middle-aged and older adults do not prepare for older age. They feel unequipped for active preparation, do not perceive the need to prepare, or they experience dependency on other parties.

These results provide important recommendations to municipalities, social welfare organizations and community health organizations in their support for middle-aged and older adults concerning age-related preparations. In turn this might lead to prevention or delay of health problems, prolonged independence and increased quality of life.

## Supplementary Information

Supplementary Material 1.

Supplementary Material 2.

## Data Availability

The datasets generated and analyzed during the current study are not publicly available due to confidentiality.

## Abbreviations

PIP: Pillar Integration Process
