# Supplementary material for: Preparing for later life in the Oosterpark neighborhood: a mixed-methods study among middle-aged and older adults
Source: BMC Public Health. 2026 Jun 17;26:2780. doi: 10.1186/s12889-026-27350-z (PMC13637094; doi:10.1186/s12889-026-27350-z)
Supplement: Supplementary file 2 [file 12889_2026_27350_MOESM1_ESM.docx]

APPENDIX 1.

Example of how the Pillar Integration Process was conducted in the study. The table presents the PIP for Theme 2: Emergencies.

.
